# BR-BCSC Signature: The Cancer Stem Cell Profile Enriched in Brain Metastases that Predicts a Worse Prognosis in Lymph Node-Positive Breast Cancer

**DOI:** 10.3390/cells9112442

**Published:** 2020-11-09

**Authors:** Maria Rita Dionísio, André F. Vieira, Rita Carvalho, Inês Conde, Mónica Oliveira, Madalena Gomes, Marta T. Pinto, Pedro Pereira, José Pimentel, Cristiano Souza, Márcia M. C. Marques, Vinícius Duval da Silva, Alison Barroso, Daniel Preto, Jorge F. Cameselle-Teijeiro, Fernando Schmitt, Ana Sofia Ribeiro, Joana Paredes

**Affiliations:** 1 Epithelial Interactions in Cancer (EPIC) group, i3S, Institute of Investigation and Innovation in Health, University of Porto, 4200-135 Porto, Portugal; medicismilenium@gmail.com (M.R.D.); avieira@ipatimup.pt (A.F.V.); rcarvalho@ipatimup.pt (R.C.); iconde@ipatimup.pt (I.C.); mcc4oliveira@gmail.com (M.O.); madalenassgomes@gmail.com (M.G.); mtpinto@i3s.up.pt (M.T.P.); fschmitt@ipatimup.pt (F.S.); aribeiro@ipatimup.pt (A.S.R.); 2 IPATIMUP- Institute of Pathology and Immunology, University of Porto, 4200-135 Porto, Portugal; 3 Centro Hospitalar de Lisboa Norte, 1649-035 Lisboa, Portugal; pedrojfp@gmail.com (P.P.); josepimentel@medicina.ulisboa.pt (J.P.); 4 In vivo CAM assays, i3S - Institute of Investigation and Innovation in Health, University of Porto, 4200-135 Porto, Portugal; 5 Department of Breast and Gynecologic Oncology, Barretos Cancer Hospital, Barretos-SP 14784-400, Brazil; crispadua10@gmail.com (C.S.); alison00wagner@gmail.com (A.B.); ddpreto@gmail.com (D.P.); 6 Molecular Oncology Research Center, Barretos Cancer Hospital, Barretos-SP 14784-400, Brazil; mmcmsilveira@gmail.com; 7 Barretos School of Health Sciences - FACISB, Barretos-SP 14784-400, Brazil; 8 Department of Pathology, Barretos Cancer Hospital, Barretos-SP 14784-400, Brazil; vinids@gmail.com; 9 Department of Pathology, Complejo Hospitalario Universitário de Vigo (CHUVI) 36204 Vigo, Spain; videoprimaria@mundo-r.com; 10 Department of Pathology, Faculty of Medicine of Porto University (FMUP), 4200-135 Porto, Portugal

**Keywords:** breast cancer, cancer stem cells, stem cell biology, prognostic factors

## Abstract

Brain metastases remain an unmet clinical need in breast oncology, being frequently found in HER2-overexpressing and triple-negative carcinomas. These tumors were reported to be highly cancer stem-like cell-enriched, suggesting that brain metastases probably arise by the seeding of cancer cells with stem features. Accordingly, we found that brain-tropic breast cancer cells show increased stem cell activity and tumorigenic capacity in the chick embryo choriallantoic membrane when compared to the parental cell line. These observations were supported by a significant increase in their stem cell frequency and by the enrichment for the breast cancer stem cell (BCSC) phenotype CD44^+^CD24^−/low^. Based on this data, the expression of BCSC markers (CD44, CD49f, P-cadherin, EpCAM, and ALDH1) was determined and found to be significantly enriched in breast cancer brain metastases when compared to primary tumors. Therefore, a brain (BR)-BCSC signature was defined (3–5 BCSC markers), which showed to be associated with decreased brain metastases-free and overall survival. Interestingly, this signature significantly predicted a worse prognosis in lymph node-positive patients, acting as an independent prognostic factor. Thus, an enrichment of a BCSC signature was found in brain metastases, which can be used as a new prognostic factor in clinically challenging breast cancer patients.

## 1. Introduction

Breast cancer is the second leading cause of brain metastases after lung cancer [1]. Of patients with metastatic breast cancer, 15–30% will develop brain metastases during the course of the disease [2]. Specifically, human epidermal growth factor receptor 2 (HER2)-overexpressing and triple-negative breast cancer (TNBC) patients are at higher risk of developing brain metastases [1,3,4,5].

Metastatic breast cancer dissemination to the central nervous system (CNS) is accompanied by neurological impairments affecting both cognitive and sensory functions, as well as an extremely poor prognosis [6]. Strategies to treat brain metastases are still very limited. Postoperative or preoperative radiation is usually delivered in conjunction with surgical resection to boost local control [7,8,9]. Further, therapies based in trastuzumab-containing regimens, bevacizumab, or small molecules inhibitors may be contemplated, as they significantly extend overall patient survival (OS) [9]. Conventional chemotherapy has shown limited activity in CNS, the culprit of which has been thought to be the blood–brain barrier along with the molecular structure of the agents [1].

Thus, to improve the management of breast cancer patients and to design prevention strategies for brain metastases, it would be highly relevant to dissect the nature of breast cancer tumor cells that are able to outgrow in the brain. Some years ago, a subpopulation of cancer cells expressing the CD44^high^CD24^−/low^ cell surface phenotype was identified in primary breast carcinomas as being highly tumorigenic and enriched for stem cell features, namely by their capacity to self-renew and to be therapy resistant [10]. Indeed, a high CD44/CD24 ratio in breast cancer has been shown as an important breast cancer prognosis predictor [11] and also to be enriched in circulating tumor cells [12,13] and in distant metastases, such as in liver [11], bone [14], and lung [15]. Interestingly, further evidence indicated that breast cancer stem cells (BCSC) are probably able to promote the metastatic cascade also to the brain [16]. McGowan and colleagues reported that Notch1 is important for the maintenance of the CD44^high^/CD24^low^ phenotype in brain-seeking cells [17], whereas Smid and colleagues showed that the expression profile of brain metastases, irrespective of the primary breast cancer subtype, displayed an upregulation of the WNT stem-like signaling pathway [18]. In addition, Sihito and colleagues demonstrated that hormone receptor-negative breast cancer patients, mainly relapsing primarily to the brain, displayed higher expression of nestin and CD133 (prominin-1) in metastases, both of which are regarded as cancer stem cell (CSC) markers of glioblastoma [19]. More recently, Sirkisoon et al. also demonstrated that TGLI1 mediates breast cancer metastasis to the brain by promoting metastasis-initiating CSCs and activating astrocytes in the brain microenvironment [20].

Aside from CD44 and CD24, other biomarkers are also considered to be able to identify and isolate BCSCs, namely, CD49f and EpCAM, which are usually used in combination to characterize cell subpopulations of the breast tissue hierarchy [21,22,23]. In particular, CD49f, also known as α6-integrin, is a BCSC marker with significant tumor-promoting signaling [24,25,26] that has been correlated with reduced breast cancer survival [27]. EpCAM is found in breast cancer circulating tumor cells [28] and it has been associated with cancer cell proliferation, survival, migration, invasion [29,30], and enhanced bone metastases’ formation [31]. Additionally, the aldehyde dehydrogenase (ALDH) superfamily of enzymes, which is involved in detoxification and/or bioactivation of various intracellular aldehydes, has been identified in both normal and malignant mammary stem cells [32]. Indeed, breast cancer cells expressing high ALDH activity contribute to metastases and chemotherapy resistance [33], and ALDH positivity in breast cancer patients has been associated with early relapse, liver metastases [11], and poor prognosis [32]. Importantly, we have also described P-cadherin as a CSC marker in basal-like breast cancer [34], being closely associated with the phenotype that identifies the luminal progenitor cell of the mammary gland [34,35]. This biomarker is an indicator of poor clinical outcome in primary breast carcinomas and lymph node metastases [36], being a putative valuable marker for axillary-based breast cancer decisions in the clinical practice [37]. Due to its strong association with invasion [38] and stem cell properties [24,34], we postulate that P-cadherin may also play a role in metastases’ colonization or growth [39].

In this work, our aim was to evaluate the stem cell profile and activity of breast cancer cells that colonize the brain, by the use of a brain-tropic breast cancer cell culture system as well as a series of human brain metastases from breast cancer patients. The main goal was to provide evidence that brain metastases are actually enriched in stem cell features. Interestingly, our data revealed a novel signature profile of five distinct BCSC markers enriched in brain metastases—the BR-BCSC signature, which is able to significantly predict poor prognosis of high-risk breast cancer patients and opens the possibility of identifying tumors with potential to metastasize to the brain.

## 2. Materials and Methods

### 2.1. Cell Culture

The breast cancer cell line MDA-MB-231 (231) was obtained from ATCC (American Type Culture Collection, Manassas, VA, USA). The human brain-tropic cell line MDA-MB-231.BR (231.Br) and MDA-MB-231.BR.HER2 overexpressing cells (231.Br.HER2) were generated as previously described [40] and were obtained from Patricia Steeg’s laboratory—NIH (National Institute of Health, Bethesda, MD, USA). The three cell lines were maintained in Dulbecco’s minimal essential media (DMEM, Invitrogen, Carlsbad, CA, USA) supplemented with 10% fetal bovine serum (FBS, Invitrogen) and with 1% antibiotic solution—penicillin streptomycin (Invitrogen). All cell lines were routinely cultured at 37 °C in a humidified atmosphere with 5% CO2. Cells were used in experiments upon reaching 70–80% confluence.

### 2.2. Cell Surface Marker Analysis by Flow Cytometry

To determine the expression of CD44, CD24, and CD49f on the cell surface of 231, 231.Br, and 231.Br.HER2 cells, we performed flow cytometry analysis. Cells were washed twice with phosphatase buffered saline (PBS) solution and then harvested with versene/0.48 mM EDTA (Invitrogen). Detached cells were washed with PBS supplemented with 0.5% FBS (stain buffer), centrifuged at 1200 rpm for 5 min, and resuspended in stain buffer. A single-cell suspension was incubated with APC-conjugated anti-CD44 (1:20) and PE-conjugated anti-CD24 (1:10), or with PerCP-Cy7-conjugated anti-CD49f (1:20). All antibodies were purchased from BD Biosciences (Temse, Belgium). Primary antibodies or the respective isotype controls (BD Biosciences) were incubated at 4 °C in the dark for 20 min. A cell viability marker was included (1:100 dilution, violet fluorescent reactive dye, Invitrogen) to discriminate dead cells. Cells were analyzed using a BD FACS Canto-II flow cytometer (BD Biosciences, Temse, Belgium).

### 2.3. Immunoblotting

Cells were lysed with PBS containing 1% Nonidet/P40 (Sigma-Aldrich, Darmstad, Germany), 1% Triton X100 (Sigma-Aldrich, Darmstad, Germany), 1:7 Protease Inhibitors Cocktail (Roche Diagnostics Gmbh, Mannheim, Germany), and 1:100 Phosphatase inhibitor (Sigma-Aldrich, Darmstad, Germany). Protein concentration was determined using the Bio-Rad protein assay (BioIRad, Richmond, CA, USA) and samples were loaded into a 10% polyacrylamide gel and transferred onto a nitrocellulose membrane (GE Healthcare Life Sciences, Sheffield, UK) at 100 V for 90 min. Membranes were blocked in 0.5% Tween 20.5% nonfat dry milk for 1 h and stained with specific primary antibodies overnight at 4 °C and with secondary antibodies for 45 min at room temperature. Detection was assessed using the ECL Chemiluminescence detection kit (Amersham Pharmacia Biotech, Piscataway, NJ, USA). Primary antibodies were anti-CD49f (dilution 1:1000, Sigma-Aldrich), anti-E-cadherin (dilution 1:1000, Cell signaling), anti-N-cadherin (dilution 1:1000, BD Transduction), anti-vimentin, anti-a-tubulin (dilution 1:1000, Sigma-Aldrich, Darmstad, Germany), and anti-GAPDH (Santa Cruz Biotechnology Inc, Heidelberg, Germany), the last of which was used as a loading control. Peroxidase-conjugated secondary antibodies (anti-rabbit and anti-mouse) were purchased from Santa Cruz Biotechnology Inc. (Heidelberg, Germany).

### 2.4. Presto Blue Assay

The 1 × 10^4^ cells were seeded in 96-well plates and incubated at 37 °C, 5% CO_2_ for 24 h. Cell viability was assessed using Presto Blue reagent: cells were washed with PBS and incubated during 30–45 min with Presto Blue solution (1:10 prepared with Presto Blue, Thermo Fisher, Whatham, MA, USA, and DMEM medium). After the incubation time, fluorescence was measured in Synergy Mx, Biotek Instruments Inc (Winooski, VT, USA) (emission: 560 nm; excitation: 590 nm).

### 2.5. Zymography Assay

The conditioned medium collected from several cell cultures, which were grown in 6-well plates coated with collagen type I, was analyzed for proteinases’ activity using gelatin and β-casein zymography. Gelatin gels were loaded with 12 μg of protein per sample and β-casein gels with 50 μg of protein per sample. Samples were mixed with sample buffer (0.03% bromophenol blue, 0.25 M Tris-HCl pH 6.8, 10% SDS (*w/v*) and 4% sucrose (*w/v*)) and electrophoresed, under nonreducing conditions, on 10% polyacrilamide gels containing 0.1% (*w/v*) gelatin or β-casein from bovine milk (Sigma-Aldrich, Darmstad, Germany). After electrophoresis, gels were washed twice, for 30 min, in 2% (*v/v*) Triton X-100 (Sigma-Aldrich, Darmstad, Germany) at room temperature, in order to remove SDS. Then they were incubated in a substrate reaction buffer for 20 h in the case of the gelatin gels (50 mM Tris-HCl, 5 mM CaCl_2_, pH 7.5) or 72 h for β-casein gels (0.2M NaCl, 5 mM CaCl2, 1% (*v/v*) Triton X-100 in 50 mM Tris-HCl, pH 7.4) and finally stained with Coomassie Blue Staining Solution (0.1% (*w/v*) Coomassie Blue R250 in 10% (*v/v*) acetic acid and 40% (*v/v*) methanol) for 25 min. The gels’ destaining was performed in a solution with 20% methanol and 10% acetic acid, until bands started to become visible. Enzymatic activity was visualized as a clear band against the blue background of stained casein gels, and matrix metalloproteases (MMPs) were identified by their molecular weight. Quantification of band density was carried out using the Quantity One software (version 4.0, BioRad, Hercules, CA, USA).

### 2.6. Mammosphere Assay

Cells were enzymatically harvested and manually disaggregated with a 25-gauge needle to form a single-cell suspension and resuspended in cold PBS. Cells were plated at 500 cm^2^ in nonadherent culture conditions, in flasks coated with 1.2% poly-(2-hydroxyethylmethacrylate)/95% ethanol (Sigma-Aldrich, Darmstad, Germany), and allowed to grow for five days in DMEM/F12 containing B27 supplement, and 500 ng/mL hydrocortisone, 40 ng/mL insulin, 20 ng/mL epidermal growth factor (EGF) in a humidified incubator at 37 °C and 5% (*v/v*) CO2. Spheres’ size was determined by a micrometer ruler used under the microscope optic. Mammospheres were considered when sphere size was superior to 60 μm of diameter. Mammosphere forming efficiency (MFE) was calculated as the number of mammospheres (≥60 μm) formed during five days divided by the original number of single cells seeded, being expressed as a percentage. Further, fold increase in mammospheres’ formation was calculated performing the ratio of the % MFE for each condition when compared with the parental cell line (231) in each biological replicate. A minimum of five independent biological experiments was performed.

### 2.7. Chicken Embryo in Vivo Growth Assay

The chicken embryo chorioallantoic membrane (CAM) model was used to evaluate the growth potential of 231, 231.Br, and 231.Br.HER2 cells. Briefly, fertilized chick (*Gallus gallus*) eggs obtained from commercial sources were incubated horizontally at 37.8 °C in a humidified atmosphere and referred to embryonic day (E). On E3, a square window was opened in the shell upon removal of 1.5–2 mL of albumin to allow detachment of the developing CAM. Consecutive cell dilutions were resuspended in 10 µL of complete medium and matrigel (1:1) (1 × 10^6^—1M, 1 × 10^5^—100K, 1 × 10^4^—10K, and 1 × 10^3^—1K cells per embryo) and placed on top of E10 growing CAM into a 3-mm silicon ring under sterile conditions (8–18 eggs per test condition/dilution). The eggs were sealed and returned to the incubator for an additional seven days. The embryos were euthanized by adding 2 mL of fixative on top of the CAM. After removing the ring, the CAM was excised from the embryos and photographed ex ovo under a stereoscope at 20× magnification (Olympus Iberia S.A.U., Barcelona, Spain, SZX16 coupled with a DP71 camera). To determine tumor area, Cells Sens software (version 1.14, Olympus Iberia S.A.U., Barcelona, Spain) was used to select and measure cell dense areas of the inoculum. According to European Directive 2010/63/EU and Portuguese law Decreto-Lei 113/2013, ethical approval is not required for experiments using embryonic chicken.

### 2.8. Limiting Dilution Assay in the in Vivo Mice Model

N:NIH(S)II-nu/nu mice strain produced at i3S, were housed, bred, and maintained at the i3S Animal House, in a pathogen-free environment, under controlled conditions of light and humidity. All the experiments were conducted with the application of the 3Rs (replacement, reduction, and refinement) (JP_2016_02 Project, animal ethics committee and animal welfare body of i3S). The groups of animals were established to use the minimal number of animals needed for a correct statistical analysis of the data.

The experiments consisted of the subcutaneous injection in the flank of female mice, with 6–8 weeks of age, with consecutive cell dilutions resuspended in matrigel (1:1) (1 × 10^6^—1M, 1 × 10^5^—100K, and 1 × 10^4^—10K, per mice) from parental 231 and brain-tropic variant 231.Br, using a 25-G needle. Mice (3–4 per group and per condition) were weighed, and tumor width and length were measured with calipers, twice a week, for a total of three weeks. Tumor volume was estimated by using the equation, V = 0.5 × a × b^2^, where V is the volume, a is the length of the major axis of the tumor, and b is the length of its minor axis.

### 2.9. Primary Breast Cancer Series

A series of 467 primary invasive breast carcinomas, diagnosed between 1978–1992, were retrieved from the Pathology Department, Hospital Xeral-Cíes, Vigo, Spain. Patient follow-up information was available for 454 cases, with a maximum follow-up of 120 months after diagnosis. The tumors were characterized for clinical and pathological features, as previously described [41]. The disease-free survival (DFS) interval was defined as the time from diagnosis to the date of breast cancer-derived relapse (DFS: mean with 95% confidence interval (CI) of 85.5 +/− 2.1 months), whereas overall survival (OS) was considered as the number of months from diagnosis to the disease-related death (OS: mean with 95% CI of 90.0 +/− 2.0 months) (Supplementary Materials Table S1). Breast cancer patients followed the adequate protocols for chemotherapy, radiotherapy, and hormone therapy given at that time. All patients were treated with adjuvant chemotherapy, which consisted of a protocol of six cycles of cyclophosphamide, methotrexate, and fluorouracil. Patients with ER-positive tumors were treated with hormonal therapy, which was carried out exclusively with tamoxifen. Patients with HER2-overexpressing carcinomas were not treated with specific targeted therapy (trastuzumab). No neo-adjuvant treatment was used in the patients included in this series. The present study was conducted under the national regulative law for the usage of biological specimens from tumor banks, where the samples are exclusively available for research purposes in the case of retrospective studies. All analyses were performed according to the REporting recommendations for tumor MARKer prognostic studies (REMARK) recommendations for prognostic and tumor marker studies.

### 2.10. Breast Cancer Brain Metastases Series

A total of 56 human brain metastases of breast cancer patients were collected in a retrospective manner from Centro Hospitalar de Lisboa Norte (CHLN), Portugal (*n* = 29), and from Barretos Cancer Hospital, Brazil (*n* = 27). Metastases present in the brain of breast cancer patients were collected after surgical resection or postmortem, preserved and fixed in formalin, included in paraffin, and carefully evaluated by neuropathologists. Clinical and pathological features were retrieved for this study. At diagnosis, the majority of patients were stage IV (26.8%, 15/56), followed by stage III (19.6%, 11/56), stage II (26.7%, 15/56), stage I (14.3%, 8/56) and for 7 patients we did not have information on the stage. Information regarding prior metastatic dissemination to the brain was not complete and it was not used in the analysis. Whenever data were available, patients were grouped into intrinsic molecular subtypes: luminal (positive for estrogen receptor (ER) and/or progesterone receptor (PgR), HER2-negative), HER2-positive (HER2-positive/amplified, negative, or positive for ER and PgR), and triple negative (ER- and PgR-negative, HER-2-negative) (Supplementary Materials Table S2). Patient follow-up information was available for 60 patients, who were diagnosed between 2009–2013 (Centro Hospitalar de Lisboa Norte) and between 2007–2008 (Barretos Cancer Hospital), with a maximum follow-up of 223 months after diagnosis. These breast cancer patients followed the established protocols of chemotherapy, radiotherapy, and hormone therapy at that time. No neo-adjuvant treatment had been used in the patients included in this series. The brain metastases-free survival (BMFS) interval was defined as the time from diagnosis to the date of breast cancer-derived brain metastases’ relapse, whereas OS was considered as the number of months from diagnosis to the disease-related death. BMFS ranged from 0–222 months (mean with 95% CI of 37.0 +/− 4.8 months and median of 25.0), whereas the OS ranged from 1–223 months (mean with 95% CI of 55.63 +/− 5.0 months and median of 42.0) (Supplementary Materials Table S2). These parameters are consistent with the biological and clinical behavior of such an aggressive disease condition. The present study was conducted with the approval of the Ethical Commission from both hospitals, under the national regulative law for the usage of biological specimens from tumor banks, where the samples are exclusively available for research purposes in retrospective studies (Ethical approvals: Barretos Cancer Hospital/Fundacao Pio XII (2-777-372) and CHLN/Nova Medical School (01/2017/CEFCM).

### 2.11. Immunohistochemistry

Immunohistochemistry for CD44, CD49f, P-cadherin, EpCAM, and ALDH1 was performed in 3-μm sections. Slides were placed in a Clear-Rite bath (Thermo Fisher Scientific, Waltham, MA, USA), rehydrated through a descending series of ethanol washes, and finally placed in distilled water. Epitope exposure was performed for 30 min at 95 °C with Tris/EDTA (Novocastra, Newcastle, UK) for CD49f and P-cadherin or citrate buffer (ThermoScientific, Freemont, CA, USA) for CD44, EpCAM, and ALDH1. Expression analyses were evaluated as follows: CD44 was detected using the antibody from Cell Signaling Technology (clone 156-3C11; Cell Signaling Technology, Danvers, MA, USA) (dilution 1:100); CD49f was assessed using the specific antibody from Sigma-Aldrich (HPA012696, Sigma-Aldrich, Darmstad, Germany) (dilution 1:50); P-cadherin expression was evaluated with the monoclonal antibody from BD Biosciences (clone 56, BD) (dilution 1:50); EpCAM was evaluated with the antibody from Santa Cruz Biotechnology (clone C-10, Santa Cruz Biotechnology, Dallas, TX, USA) (dilution 1:50); and ALDH1 was detected with an antibody from Abcam (clone EP1933Y, Abcam, Cambridge, UK) (dilution 1:100). Primary antibodies were detected using a secondary antibody with horseradish peroxidase polymer (Cytomation Envision System HRP; DAKO, Carpinteria, CA, USA) (CD49f, P-cadherin, EpCAM, ALDH1) or with the labeled biotin-streptavidin method (DAKO, Carpinteria, CA, USA) (CD44), according to the manufacturer’s instructions. The detection method used diaminobenzidine (DAB) as chromogen. Of note, the BCSC markers CD44, CD49f, P-cadherin, and ALDH1 were previously characterized in the primary breast cancer series [34,41,42]; thus, only the EpCAM marker was performed in this specific series, as described. For breast cancer brain metastases, all BCSC markers were evaluated in the context of this work.

### 2.12. Immunohistochemical Evaluation

The expression of CD44, CD49f, P-cadherin, EpCAM, and ALDH1 was independently evaluated by one pathologist (F.S.) based on grading systems previously established [42,43,44]. Positive staining of CD44, CD49f, and P-cadherin was considered when detected at the membrane of tumor cells and the extension of scoring was considered as follows: 0, 0–10% of positive tumor cells; 1+, 10–25% of positive tumor cells; 2+, 25–50% of positive tumor cells; 3+, more than 50% of positive tumor cells. For CD44, CD49f, and P-cadherin, the cases were classified as (0) when considered negative, whereas (1+), (2+), and (3+) were determined as positive cases. For EpCAM, only membranous staining intensity was considered, with strong and moderate expression being considered positive and low/absent expression being considered negative. Regarding ALDH1, it was classified as positive when more than 1% of tumor cells showed clear cytoplasmic positivity.

### 2.13. Statistical Analysis

Mammospheres’ forming efficiency and tumor growth area in the CAM assay data were tested for significance using the Tukey’s test and Mann–Whitney test, respectively. Flow cytometry experiments were carried out three times, with data pooled from all three. For the immunohistochemistry results, contingency tables and chi-square tests to estimate the relationship between staining patterns of each BCSC marker in both primary and metastasis series were performed. Survival analyses were estimated by the Kaplan–Meier method and compared using the log-rank test or with the Cox proportional hazards model. Statistical analyses were carried out using SPSS statistics V.17.0 software package for Windows (SPSS, Inc., Chicago, IL, USA) and Graph Pad Prism version 5.0c software (Graph Pad Software, San Diego, CA, USA), unless otherwise stated. *P* values lower than 0.05 were considered statistically significant. All statistical tests were two-sided.

## 3. Results

### 3.1. Brain-Tropic Metastatic Breast Cancer Cells Show Increased Stem Cell Activity and Tumorigenic Potential

A brain-tropic metastatic breast cancer model was used. In detail, it was developed by the intra-cardiac injection of human MDA-MB-231 (231) metastatic breast cancer cells into nude mice and by the subsequent isolation of metastases from the brain and their reculture in vitro and reinjection into mice. After multiple sequential rounds of selection, a brain-tropic clone was obtained (231.Br), where cancer cells acquired a higher ability to metastasize to this specific location compared to the original parental cell line, which showed a broader metastatic pattern [40]. Additionally, these brain-seeking breast cancer cells were transfected to overexpress HER2 (231.Br.HER2) [45]. We selected these two cell models, since HER-2 overexpression is enriched in breast cancer brain metastases and, importantly, this brain-tropic variant with Her-2 overexpression induces larger brain metastases than the brain-tropic variant without HER2. These data made us speculate if the CSC potential could be further enriched in 231.Br.HER2 cells.

**Figure 1:**
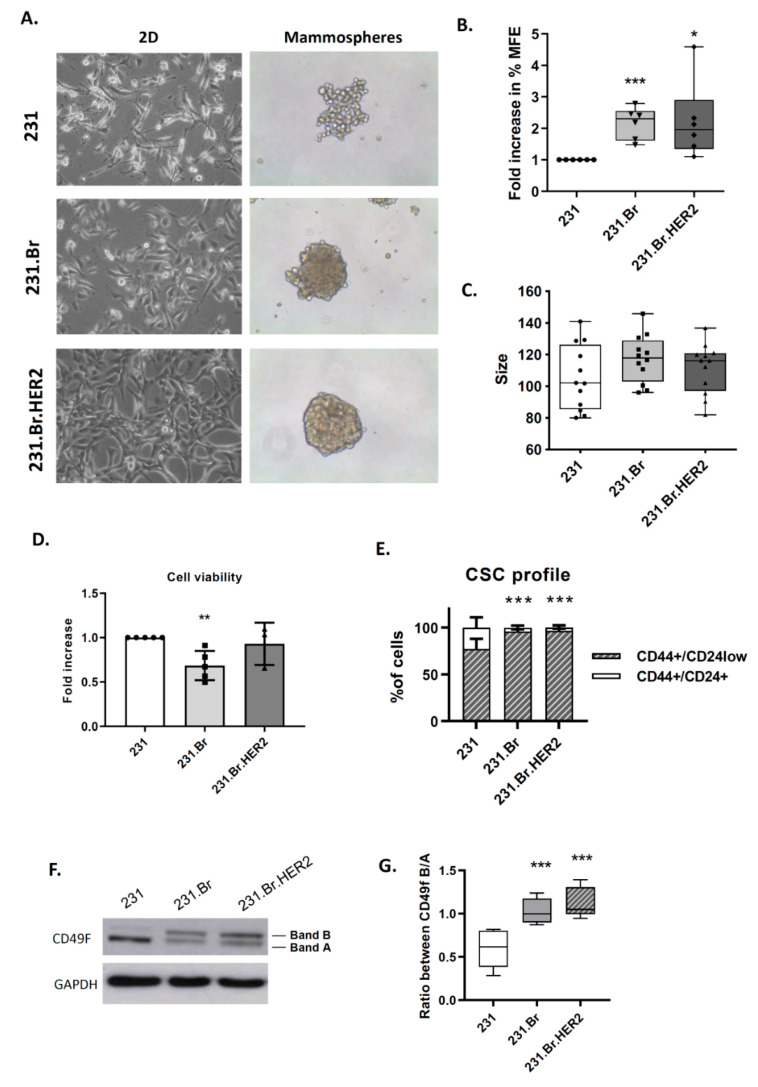
Brain-tropic metastatic breast cancer cells show increased stem cell activity. (**A**) Representative pictures of monolayer (2D) and mammospheres (20× magnification). (**B**) Fold increase in the % of mammospheres’ formation efficiency (MFE) in brain-tropic cells (231.Br and 231.Br.HER2) compared with parental cells (231). Each dot in the graph represents an independent biological replicate. Both brain-tropic cells showed to have a significant increase in the stem cell activity. (**C**) Size of the mammospheres (spheres > 60 μm) at the end of the 5-day culture. (**D**) Fold change in cell viability and metabolic activity measured by the Presto blue assay, in brain-tropic cells (231.Br and 231.Br.HER2) compared with parental cells (231). Each dot in the graph represents an independent biological replicate. (**E**) Percentage of cells with the stem cell profile CD44+/CD24−, measured by flow cytometry. (**F**) Western blot for CD49f expression detecting two specific bands (**A** and **B**). (**G**) Ratio between the upper band (**B**)/lower band (**A**), using pixel density from each band. In all assays 4 to 6 biological replicas were performed. Statistical analysis * *p* < 0.05, ** *p* < 0.01, *** *p* < 0.001.

Interestingly, we found that both brain-tropic cells were morphologically different from parental cells when grown in 2D, as well as when grown in suspension, indicating their putative enrichment in stem cell features (Figure 1A and Supplementary Materials Figure S1A).

We performed the mammospheres’ assay to measure their ability to grow in anchorage-independent conditions, which revealed that both cell lines with tropism to the brain yielded larger mammospheres with a compact and epithelial-like spherical structure, whereas the parental cell line exhibited mammospheres with a loose, grape-like and more mesenchymal-like structure and no central compact aggregate (Figure 1A). These morphological differences in cell phenotype were further confirmed by Western blot. Although no expression of E- and N-cadherin was detected in any cancer cell model, a decrease in vimentin expression was observed in the brain-tropic breast cancer cells (Supplementary Materials Figure S1B). Interestingly, we also found deregulation of MMPs activity when brain tropic cells were compared with the parental cell line (decrease in MMP9 and increase in MMP1 (Supplementary Materials Figure S1C). Aside from the morphological differences, the number and the percentage of mammospheres’ forming efficiency (MFE) of brain-tropic cells showed to be significantly increased when compared to the parental cell line (Figure 1B and Supplementary Materials Figure S2A), while no differences were observed in the size of the mammospheres (Figure 1C) or on cell viability (Figure 1D). Interestingly, the stem-like phenotype was further confirmed by the enrichment in the CD44^+^CD24^−/low^ cell surface expression. All cell lines were constituted by a large subpopulation of CD44^+^/CD24^−^ cells; but a small, but very significant, enrichment in CD44^+^/CD24^−^ subpopulations was found in brain-tropic cells when compared with the parental cell line (with 76.9% in 231, 95.7% in 231.Br, and 96.4% in 231.Br.HER2 cells) (Figure 1E and Supplementary Materials Figure S3A,B). We also evaluated the expression of CD49f by Western blot, since two different isoforms with different functions in stemness have been reported [46]. Interestingly, we observed that the parental cell line and brain-tropic breast cancer cells displayed a different profile of these bands: a significant increase was observed in the ratio between high molecular weight (MW) band (band B) and low MW band (band A) (ratio B/A) in brain-tropic cells when compared with the parental cell line, which also supports that brain-tropic cells are enriched in stem cell activity [46] (Figure 1F,G).

**Figure 2:**
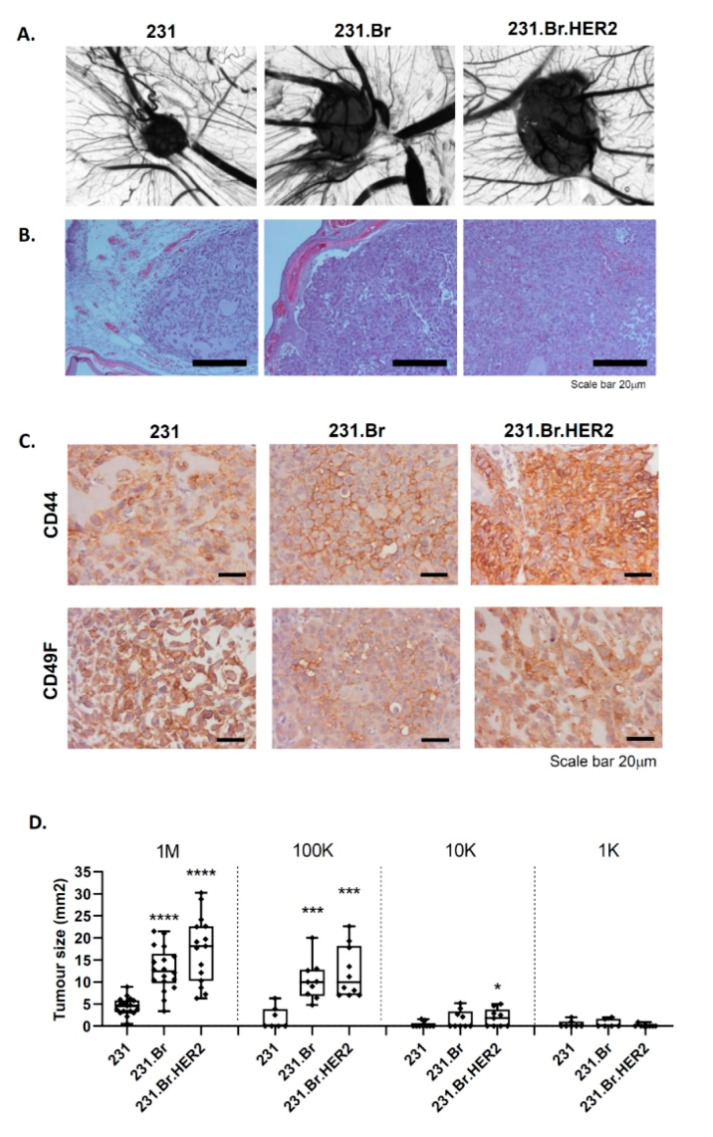
Brain-tropic metastatic breast cancer cells display an increased tumorigenic ability in the choriallantoic membrane of the chick embryo (CAM). (**A**) Representative pictures of tumors formed in the CAM, taken under a stereomicroscope (4× magnification). (**B**) Hematoxilin-eosin (HE) staining of the CAM tumors. Scale bar = 20 μm. (**C**) Illustrative representation of the immunohistochemical evaluation of the breast cancer stem cell markers CD44 and CD49f in CAM tumors. Scale = 20 μm. (**D**) Tumor size evaluation in a limiting dilution assay performed in the CAM, by inoculation of 1M, 100K, 10K, and 1K cell number per egg. Each dotted point corresponds to an independent biological replicate (1M: 18, 18, 15 eggs; 100K: 9, 10, 10 eggs; 10K: 10, 10, 10 eggs; and 1K: 8, 10, 9 eggs for 231, 231.Br, and 231.Br.HER2 respectively). Statistical analysis * *p* < 0.05, *** *p* < 0.001, **** *p* < 0.001).

Finally, we tested the ability of brain-tropic cells to grow and form in vivo tumors in the chick embryo chorioallantoic membrane (CAM) and in immunocompromised mice. All cell lines inoculated in the CAM were able to form tumors; however, brain-tropic variants formed significantly larger and more compact tumors than those formed by the parental cell line, which typically formed more disaggregated and diffuse tumors (Figure 2A,B).

Interestingly, an enrichment of CD44 expression was found in brain-tropic CAM tumors, whereas no significant differences were observed for CD49f expression (Figure 2C). Additionally, a limiting dilution assay was performed to evaluate the stem cell frequency of brain-tropic breast cancer cells. We evaluated not only the tumor size, but also the frequency of tumor formation. As shown in Figure 2D, both 231.Br and 231.Br.HER2 cells formed significantly bigger tumors when 1 M and 100K cells were inoculated in the CAM. Moreover, although tumor size differences were not maintained when 10K and 1K cells were inoculated, a significant impact was still observed in the frequency of tumor formation, as shown in Table 1. This same result was observed for the tumors formed in the immunocompromised animals (Supplementary Materials Figure S2B,C). In fact, statistical analysis, using the ELDA software [47], revealed a significant increase in the stem cell frequency of the brain-tropic breast cancer cells (*p* < 0.0001), although no significant differences were observed between 231.Br and 231.Br.HER2.

### 3.2. Human Breast Cancer Brain Metastases Are Enriched in BCSC Markers

**Figure 3:**
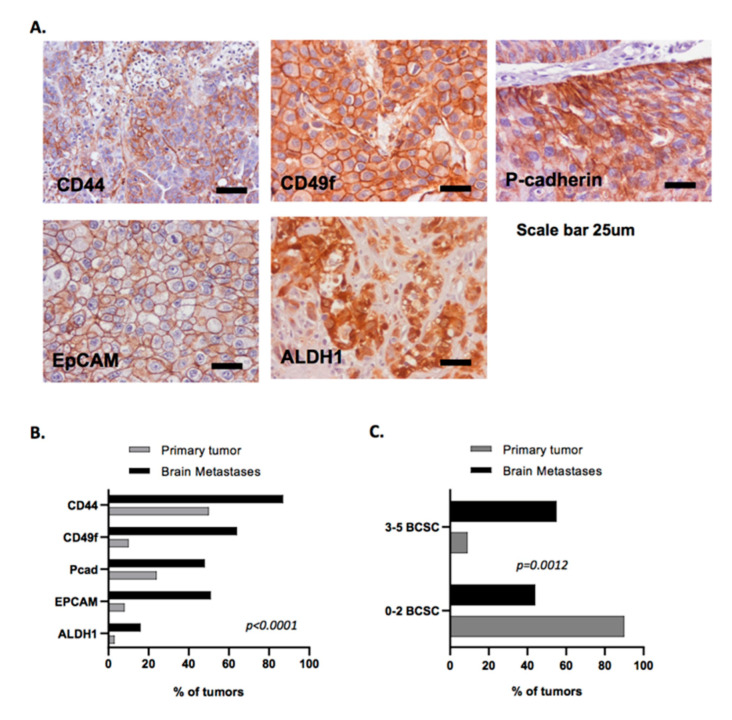
Human breast cancer brain metastases are enriched in breast cancer stem cell markers. (**A**) Illustrative representation of the immunohistochemical evaluation of the breast cancer stem cell markers CD44, CD49f, P-cadherin, EpCAM, and ALDH1 in human brain metastases. Scale = 25 μm. (**B**) Percentage of positive tumors with expression for each BCSC marker (CD44, CD49f, P-cadherin, EpCAM, and ALDH1) in the breast cancer brain metastases series vs. primary breast cancer patients. (**C**) Percentage of positive tumors with BR-BCSC signature (expression of any combination of 3–5 BCSC) in the breast cancer brain metastases series vs. primary breast cancer patients. Associations between the expression of the markers evaluated in primary tumors and breast cancer brain metastases were assessed by Pearson’s *χ*2.

Based on our observations in brain-tropic breast cancer cell lines, as well as on literature that demonstrate that CSCs have a pivotal role in tumor growth and metastases [48], we decided to study the expression of five BCSC biomarkers in a series of human breast cancer brain metastases and compare it with the expression of these same proteins in a large series of primary breast cancers. Thus, we evaluated the expression of CD44 and CD49f, as well as P-cadherin, EpCAM, and ALDH1 [41,42,43]. CD44, CD49f, and P-cadherin expression were mainly membranous with some cytoplasmic staining. EpCAM expression was mainly membranous and ALDH1 expression was found essentially in the cytoplasm. A representative staining of the immunohistochemistry profile is shown in Figure 3A.

As shown in Figure 3B (and Supplementary Materials Tables S3 and S4), we compared the expression of each BCSC marker between unpaired primary breast carcinomas and breast cancer brain metastases. Interestingly, all BCSC markers showed to be significantly enriched in brain metastases when compared with primary tumors (Supplementary Materials Table S5). CD44, CD49f, P-cadherin, EpCAM, and ALDH1 were expressed in 87.7% vs. 51.2%, 66.7% vs. 11.5%, 50.9% vs. 24.5%, 52.6% vs. 9%, and 17% vs. 3% of brain metastases vs. primary tumors, respectively. In summary, we found an overall stem cell enrichment in human brain metastases, supporting the observations made in brain-tropic cell lines.

### 3.3. BCSC Signature Enriched in Brain Metastasis Is Significantly Associated with a Patient’s Worse Prognosis

Numerous reports have shown the role of CSC markers in the maintenance of stem cell properties in tumor cells by their impact in cancer initiation and invasion. However, few studies evaluated the impact of a defined CSC enriched profile in breast cancer progression and metastases. Thus, we defined a brain BCSC (BR-BCSC) signature, encompassing the expression of 3–5 of the previously evaluated BCSC markers in brain metastases, in order to explore its prognostic impact in terms of survival for breast cancer patients. Interestingly, and in accordance with the previously observed enrichment of the distinct BCSC markers in the brain metastases series, we found a very significant enrichment of the BR-BCSC signature in breast cancer brain metastases when compared with primary tumors (55.6% vs. 9.3% in primary tumors for 3–5 BCSC, Figure 3C).

After, we compared the impact of each BCSC marker alone with the BR-BCSC signature (0–2 BCSC vs. 3–5 BCSC markers) on patients’ survival, when evaluated in both series of primary carcinomas and brain metastases. As previously reported, P-cadherin was the only marker that showed an impact on DFS and OS for breast cancer patients, when evaluated in the primary breast carcinomas (Supplementary Materials Table S6) [41]. Interestingly, CD44 was the only breast CSC marker with impact in brain metastases-free survival prognosis (BMFS), showing a decrease from 68.5 ± 21.8 months to 33.4 ± 4.5 months (*p =* 0.029) (Table 2) when its expression was evaluated in brain metastases.

**Figure 4:**
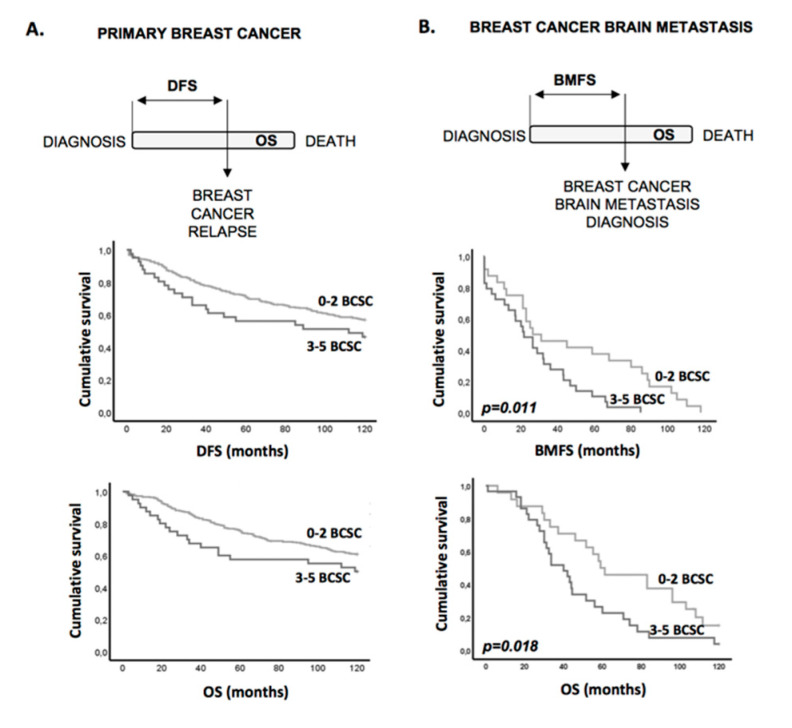
The BR-CSC signature associates with worse prognosis. (**A**) Kaplan–Meier analysis of disease-free survival (DFS) and overall survival (OS) using the BR-BCSC signature in the primary breast cancer series. Five-year DFS (50.2 ± 0.9 months to 43.6 ± 3.3 months, *p* = 0.018) and 5-year OS (52.8 ± 0.8 months to 45.1 ± 3.2 months, *p* = 0.004). (**B**) Kaplan–Meier analysis of brain metastases-free survival and overall survival using the SC enriched profile in the breast cancer brain metastases series. Ten-year brain metastasis free survival (BMFS) (48.1 ± 8.1 months to 26.7 ± 4.3 months, *p* = 0.008) and OS (68.8 ± 7.6 months to 46.2 ± 5.4 months, *p* = 0.02).

None of the other BCSC markers evaluated had an impact on DFS, BMFS, or OS of breast cancer patients (Table 2 and Supplementary Materials Table S6). However, when we grouped the patients according to the defined BR-BCSC signature, we could observe a significant association with a decrease in both 5-year DFS (50.2 ± 0.9 months to 43.6 ± 3.3 months, *p =* 0.018) and 5-year OS (52.8 ± 0.8 months to 45.1 ± 3.2 months, *p* = 0.004) when evaluated in the primary breast cancer series (Supplementary Materials Table S6 and Figure 4A), as well as with a worse 10-year BMFS (48.1 ± 8.1 months to 26.7 ± 4.3 months, *p* = 0.008) and OS (68.8 ± 7.6 months to 46.2 ± 5.4 months, *p* = 0.02) when evaluated in breast cancer brain metastases (Table 2 and Figure 4B).

Interestingly, the BR-BCSC signature was associated with clinico-pathological features associated with poor prognosis, such as increased tumor size and high histological grade, but no association was found with regional lymph node involvement (Supplementary Materials Table S7). Furthermore, this BCSC enriched phenotype was inversely correlated with ER and PgR expression, but positively and significantly associated with Ki-67, EGFR, vimentin, CK5, CK14 and p63 (Supplementary Materials Table S8), as well as with the metabolic markers GLUT 1 and CAIX (Supplementary Materials Table S8).

Altogether, these results indicate that BCSC enriched tumors strongly associate with aggressive behavior features, such as high proliferation rates, poor differentiation, and basal-like and glycolytic profiles. Actually, univariate analysis showed that the BR-BCSC signature significantly impacts on the prognosis of breast cancer patients (5-year DFS: hazard ratio (HR) = 1.807, *p =* 0.021; 5-year OS: HR = 2.090, *p =* 0.005). However, this impact was lost in a multivariate Cox regression analysis, including the classical breast cancer prognostic factors (Supplementary Materials Table S9), showing that it is not an independent feature.

### 3.4. BR-BCSC Signature Strongly Predicts a Poor Overall Survival in Lymph Node-Positive Breast Cancer Patients

**Figure 5:**
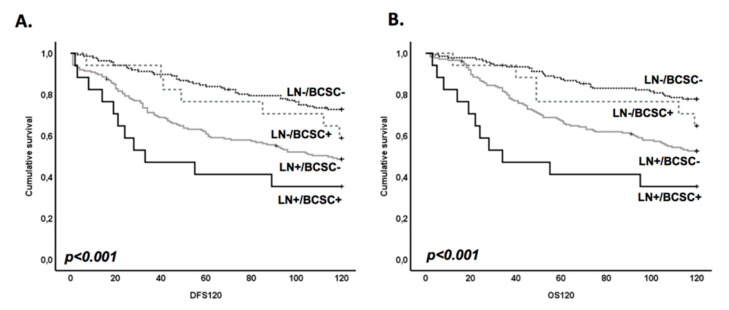
BR-BCSC signature strongly predicts a poor overall survival in lymph node-positive breast cancer patients. Kaplan–Meier analysis of DFS (**A**) and OS (**B**) using the BR-BCSC signature in both lymph node-positive and lymph node-negative breast cancer. LN-positive cases with a BR-BCSC signature were from patients who exhibited the worst DFS (59.8 ± 11.8 months vs. 79.0 ± 3.5 months in LN-positive cases with no BR-BCSC signature) and OS (60.4 ± 11.8 months vs. 86.2 ± 3.2 in LN-positive cases with no BR-BCSC signature).

As the BR-BCSC signature was not associated with the lymph node (LN) involvement, which is one of the major prognostic factors in breast cancer, we decided to combine these two parameters in order to investigate whether the variation in BR-BCSC signature could allow the stratification of LN-positive patients regarding their clinical behavior. Remarkably, we found that breast cancer LN-positive cases with a BR-BCSC signature were from patients exhibiting the worst disease-free survival (59.8 ± 11.8 months vs. 79.0 ± 3.5 months in LN-positive cases with no BR-BCSC signature) and overall survival (60.4 ± 11.8 months vs. 86.2 ± 3.2 in LN-positive cases with no BR-BCSC signature) (Figure 5A,B and Supplementary Materials Table S6), in comparison with other combinatorial possibilities. Accordingly, cases scored as harboring a BR-BCSC positive in the primary tumor, but for which lymph nodes were classified as negative, showed a better prognosis (Figure 5 and Supplementary Materials Table S6, *p* < 0.001 for DFS and OS).

Univariate Cox regression analysis showed that the BR-BCSC signature in the LN-positive patients was the most relevant factor in predicting poor prognosis, with a HR of 5.708 for 5-year DFS (*p* < 0.001) and a HR of 3.802 for 10-year DFS (*p* < 0.001), when compared with tumors remaining negative in the metastatic axillary site (Table 3). The same significant result was observed for OS (HR = 7.894, *p* < 0.001 at 5-year OS; HR = 5.101, *p* < 0.001 at 10-year OS) (Table 3). Importantly in this case, the multivariate analysis showed that the effect of the BR-BCSC signature was independent of primary tumor size and histological grade (HR = 3.490, *p* = 0.002 for 5-year DFS; HR = 4.133, *p* = 0.001 for 5-year OS; HR = 2.335, *p* = 0.019 for 10-year DFS; HR = 3.042, *p* = 0.003 for 10-year OS) (Table 3).

Therefore, for the first time, we have demonstrated that a BCSC signature, which is enriched in brain metastasis, strongly predicts a poor prognosis in lymph node-positive breast cancer patients.

## 4. Discussion

Distant metastases’ formation is a complex process, and the most limiting step is the outgrowth of tumor cells at distant sites [49]. According to the cancer stem cell theory, cancer cells are considered to exist in different populations, some of which may have stem cells’ properties, such as self-renewal capacity, that may aid their adaptation to distant organs with distinct microenvironments. Although various CSC markers have been widely used to characterize stem properties of cancer cells and to predict patient prognosis, few studies investigate the dynamics of CSC markers’ expression during the brain metastatic cascade.

In order to understand the role of CSCs in breast cancer brain metastases, we took advantage of the MDA-MB-231 breast cancer metastatic cell model in order to evaluate if there was an enrichment of stem-like properties in breast cancer cells with organotropism to the brain. In particular, we tested two brain-tropic variants, with and without HER-2 overexpression, previously established and characterized in terms of metastatic potential [45], since Her-2 overexpression may affect the natural history of breast cancer to accelerate brain progression.

Additionally, we also characterized the expression of five known BCSC markers, namely, CD44, CD49f, P-cadherin, EpCAM, and ALDH1 [24,34], in brain metastatic tumor samples in order to compare it with the expression found in primary tumors. Of note, Lawson and colleagues performed single-cell analysis of primary and metastatic tissues and found that brain metastatic cells show a distinct gene expression signature, with the highest levels of stem cell, quiescence, and anti-apoptosis genes found in brain metastases [35].

In this study, we found that brain-tropic breast cancer cells have an enrichment in their stem-like profile, since they display increased MFE, form larger tumors when inoculated in the CAM, and show a higher percentage of the CD44^+^/CD24^−/low^ population. In a study using the same cell model, McGowan and colleagues reported only a marginal difference between parental and brain-tropic cells regarding the in vitro CD44^+^/CD24^−^ phenotype [17], although they showed that this CSC population was enriched in metastases from mice. Furthermore, the authors also demonstrated that inhibition of a stem cell signaling pathway, specifically Notch signaling, in brain-tropic cells reduced the expression of the CD44^hi^/CD24^low^ phenotype and decreased metastases’ formation [17].

Interestingly, we confirmed the increased stem cell potential of the brain-tropic cells by performing limiting dilution assays using the ex vivo CAM model. In fact, we observed a significant increase in the stem cell frequency of cells that show tropism to the brain when compared with the parental cells. These results corroborate a recent study where the authors show that targeting brain adaptive CSC signaling inhibits the brain metastatic colonization [50].

The brain-tropic breast cancer cells did not only show increased stem cell potential but also showed a striking difference in their phenotype, being more cohesive and exhibiting a more epithelial-like phenotype, in contrast to parental cells that displayed a mesenchymal morphology. Interestingly, we found an altered CD49f protein expression pattern between these cells, where brain-tropic cells yielded two bands, as detected by Western blot analysis, most probably corresponding to the two different isoforms previously reported to be associated with different stem cell-related functions. CD49f isoform B differs from isoform A due to the addition of a sequence of 18 amino acids to the cytoplasmic domain, promoting the regulation of stem cell responses to biochemical stimuli and/or biophysical cues in the stem cell niche, thus impacting the stem cell fate [46]. Interestingly, an increase was observed in the ratio of the stem cell-related isoform B with isoform A, suggesting an important role of this isoform on the stem ability of brain-tropic breast cancer cells.

In accordance, we also found a significant enrichment of the BCSC markers CD44, CD49f, P-cadherin, EpCAM, and ALDH1 in human breast cancer brain metastasis when compared with unmatched primary tumors previously analyzed by our group [34,41,42,51]. This clearly indicates that there is an imbalance of adhesion/stem cell molecules that lead to a modification in malignant and colonization properties in distant sites, such as the brain [18,52,53,54,55,56,57]. However, more importantly, our study revealed an enriched stem cell profile defined by the simultaneous expression of 3–5 of these BCSC markers, which we defined as a BR-BCSC signature. Even though only 9.3% of the primary tumors display the BR-BCSC signature, these were found to be significantly associated with poor clinico-pathological features and with aggressive behavior properties, such as high proliferation rates, poor differentiation, basal-like features, and glycolytic profiles. These results are in accordance with data showing that predisposition or adaptation of the tumor cell energy metabolism is a key element in breast cancer brain metastases [58] and raises the possibility of targeting the functional differentiation of breast cancer brain metastases in therapeutic strategies.

Remarkably, patients with tumors enriched for the BR-BCSC signature exhibited a significant decrease in the 5-year DFS and OS when evaluated in primary breast cancer series, as well as in the 10-year BMFS and OS in the breast cancer brain metastases series, indicating that this profile would impact on the prognosis of breast cancer patients. Although not acting as an independent prognostic factor, our data disclose, for the first time, a signature of BCSC markers that significantly associates with poor prognostic tumors and that could probably help to identify tumors with potential to metastasize to the brain. Zhang and collaborators have already also suggested a four-protein signature (brain metastases selected markers (BMSM)) to identify CTCs with potential to metastasize to the brain, demonstrating that HER2+/EGFR+/HPSE+/Notch1+ circulating tumor cells (CTCs) displayed a significant propensity to metastasize to the brain in contrast to the parental CTC cells [59]. Although, targeting or isolating CTCs for clinical purposes still remains a challenge, it would be further interesting to test if our BR-BCSC signature would be able to isolate/identify CTCs that specifically metastasize to the brain.

Ultimately, the great novelty of our study was to demonstrate that we can use the BR-BCSC signature as an independent prognostic factor in LN-positive breast carcinomas. In this specific setting, we could observe that tumors harboring a BR-BCSC signature have a very significant and increased risk to recur after five years of the breast cancer diagnosis, as well as to die from this disease. These results are important to be clinically taken into account, since there are few biomarkers described until now that can be useful to stratify this group of high-risk breast cancer patients. In 2006, Brennan et al. also showed that CAIX expression did not correlate with lymph node status but was associated with a significantly worse prognosis in breast cancer patients harboring 1–3 positive lymph nodes. Cox multivariate analysis of the patients in the control arm of the trial revealed CAIX expression to be an independent prognostic factor of breast cancer-specific death in this subgroup [60]. This kind of knowledge is important to be acquired, since the involvement of lymph nodes is used to define patients who will require axillary radiotherapy and to determine the type and aggressiveness of adjuvant systemic therapy. Thus, the data presented in this study demonstrate that the BR-BCSC signature is a marker of poor prognosis in LN-positive patients, but probably can also be a marker of therapy resistance. However, to reliably measure its effect on therapy resistance, further studies are needed with cohorts specifically selected to address this question.

In summary, our data demonstrate that there is an enrichment of a stem-like profile in human brain metastasis, which is associated with a worse breast cancer prognosis. In particular, we found that this BR-BCSC signature is a prognostic factor for DFS and OS in patients with node-positive breast cancer. Thus, the characterization of this signature should be added as an independent prognostic variable and used to predict breast cancer patients at risk, helping in defining an improved and targeted therapy to this aggressive disease condition.

## Figures and Tables

**Table 1:** Number of tumors formed in the different cell lines using serial dilutions of inoculated cells (limiting dilution assays). 8–18 independent biological replicates have been performed in the different conditions (1M: 18, 18, 15 eggs; 100K: 9, 10, 10 eggs; 10K: 10, 10, 10 eggs and 1K: 8, 10, 9 eggs for 231, 231.Br and 231.Br.HER2 respectively). Using the ELDA software (http://bioinf.wehi.edu.au/software/elda/), a stem cell frequency was determined. A significant difference was obtained between the parental 231 and the brain-tropic variants (231.Br and 231.Br.HER2), however no significant enrichment is observed for 231.Br vs. 231.Br.HER2.

Cell Model	1M	100K	10K	1k	Stem Cell Frequency (ELDA)	*p*-Value (vs 231) *	*p*-Value **
**231**	18/18 (100%)	5/9 (55.6%)	2/10 (20%)	3/8 (37.5%)	1/68769	-	7.31 × 10^−5^
**231.Br**	18/18 (100%)	10/10 (100%)	4/10 (40%)	3/10 (30%)	1/12215	0.0007	
**231.Br.HER2**	15/15 (100%)	10/10 (100%)	6/10 (60%)	1/9 (11.1%)	1/10535	0.0003	

Multiple group statistical analysis: * Overall test for differences in stem cell frequencies between any of the groups; ** Pairwise tests for differences in stem cell frequencies.

**Table 2:** Survival for the individual breast cancer stem cell (BCSC) markers CD44, CD49f, P-cadherin, EPCAM, and ALDH1 and for the brain (BR) BCSC (BR-BCSC) signature. Survival means were estimated using the Kaplan–Meier method and compared using the log-rank (Mantel–Cox) test. A significant level of 5% was considered. Missing cases were not considered for statistical analysis. Mean survival are in bold as well as the significant *p*-values.

	Breast Cancer Brain Metastases									
	Brain Metastasis Free Survival					Overall Survival				
	10-Year BMFS					10-Year OS				
	Mean	Std dev	95% CI		*p*-Value	Mean	Std dev	95% CI		*p*-Value
			Inferior	Superior				Inferior	Superior	
CD44										
Negative (n = 7)	**68.5**	21.8	25.8	111.3	**0.029**	**81.5**	16.0	50.1	112.8	**0.045**
Positive (n = 49)	**33.4**	4.5	24.6	42.2		**53.4**	4.7	44.2	62.6	
CD49F										
Negative (n = 19)	**41.9**	10.5	21.2	62.6	0.552	**61.2**	8.7	44.2	78.1	0.488
Positive (n = 37)	**35.6**	5.2	25.4	45.9		**54.6**	5.6	43.7	65.6	
P-cadherin										
Negative (n = 27)	**41.6**	7.1	27.6	55.6	0.144	**64.7**	7.1	50.8	78.6	0.067
Positive (n = 27)	**30.5**	5.4	19.9	41.1		**47.9**	5.9	36.4	59.4	
EPCAM										
Negative (n = 27)	**46.8**	8.3	30.6	63.1	0.07	**65.0**	7.3	50.7	79.2	0.091
Positive (n = 29)	**29.3**	5.3	19.0	39.6		**49.5**	5.8	38.1	60.9	
ALDH1										
Negative (n = 57)	**33.8**	4.1	25.8	41.8	0.313	**53.8**	4.4	45.1	62.5	0.535
Positive (n = 9)	**53.4**	23.1	8.2	98.6		**55.7**	12.9	30.4	81.0	
BR-BCSC										
0–2 BCSS markers (n = 24)	**48.1**	8.1	32.1	64.0	**0.008**	**68.8**	7.6	54.0	83.7	**0.02**
3–5 BCSS markers (n = 29)	**26.7**	4.3	18.2	35.1		**46.2**	5.4	35.6	56.8	

**Table 3:** Univariate and multivariate Cox proportional hazard analysis for the lymph-node (LN) status and BR-BCSC signature in the primary carcinomas. This analysis allows risk prediction (hazard ratios and the corresponding 95% confidence interval) for DFS and OS of breast cancer patients, in the primary tumor series. A significant level of 5% was considered. Missing cases were not considered for statistical analysis. The multivariate Cox regression analysis included the effects of histological grade and tumor size.

		Disease-Free Survival								Overall Survival							
	Primary Tumor	5-Year Survival				10-Year Survival				5-Year Survival				10-Year Survival			
		HR	95% CI		*p*-Value	HR	95% CI		*p*-Value	HR	95% CI		*p*-Value	HR	95% CI		*p*-Value
			Inferior	Superior			Inferior	Superior			Inferior	Superior			Inferior	Superior	
Univariate analysis	LN/BR-BCSC																
	Negative/Negative (*n* = 136, ref.)	1.00			—	1.00			—	1.00			—	1.00			—
	Negative/Positive (*n* = 17)	1.62	0.55	4.75	0.38	1.57	0.70	3.52	0.27	2.08	0.69	6.21	0.19	1.68	0.70	4.04	0.25
	Positive/Negative (*n* = 174)	2.94	1.78	4.85	0.00	2.36	1.61	3.46	0.00	3.13	1.79	5.45	0.00	2.60	1.71	3.95	0.00
	Positive/Positive (*n* = 17)	5.71	2.67	12.21	0.00	3.80	1.94	7.46	0.00	7.89	3.58	17.42	0.00	5.10	2.55	10.19	0.00
Multivariate analysis	LN/BR-BCSC																
	Negative/Negative (*n* = 136, ref.)	1.00			—	1.00			—	1.00			—	1.00			—
	Negative/Positive (*n* = 17)	1.16	0.39	3.49	0.79	1.20	0.52	2.74	0.67	1.35	0.44	4.12	0.60	1.25	0.51	3.07	0.62
	Positive/Negative (*n* = 174)	2.33	1.35	4.02	0.00	1.89	1.25	2.85	0.00	2.15	1.19	3.88	0.01	1.98	1.27	3.10	0.00
	Positive/Positive (*n* = 17)	3.49	1.56	7.83	0.00	2.34	1.15	4.73	0.02	4.13	1.80	9.51	0.00	3.04	1.47	6.29	0.00
	Histological grade																
	Grade I (*n* = 54, ref.)	1.00			—	1.00			—	1.00			—	1.00			—
	Grade II (*n* = 94)	0.91	0.39	2.13	0.84	1.21	0.63	2.32	0.56	1.11	0.43	2.86	0.83	1.19	0.61	2.34	0.61
	Grade III (*n* = 175)	1.85	0.87	3.92	0.11	1.85	1.02	3.37	0.04	2.00	0.85	4.71	0.11	1.62	0.87	3.02	0.13
	Tumor size																
	T1: <2 cm (*n* = 82, ref.)	1.00			—	1.00			—	1.00			—	1.00			—
	T2: 2–5 cm (*n* = 189)	1.86	0.93	3.73	0.08	1.65	0.98	2.77	0.06	2.70	1.13	6.43	0.03	1.88	1.06	3.35	0.03
	T3: >5 cm (*n* = 52)	3.21	1.49	6.91	0.00	2.99	1.66	5.39	0.00	5.11	2.02	12.92	0.00	3.45	1.81	6.59	0.00

## Supplementary Materials

The following are available online at https://www.mdpi.com/2073-4409/9/11/2442/s1. Supplementary Figure S1. Epithelial Mesenchymal phenotype. Supplementary Figure S2: CSC activity, Supplementary Figure S3: Representative flow cytometry analysis data, Supplementary Table S1: Characterization and clinico-pathological features of the primary breast cancer series, Supplementary Table S2: Characterization and clinico-pathological features of the two brain metastases series (from Portugal and Brazil), Supplementary Table S3: Frequency of expression of the BCSC markers CD44, CD49F, P-cadherin, EPCAM, and ALDH1 and the BR-BCSC signature in independent breast cancer brain metastases, Supplementary Table S4: Frequency of expression of the BCSC markers CD44, CD49F, P- cadherin, EPCAM and ALDH1 the BR-BCSC signature in primary carcinomas, Supplementary Table S5: Contingency table with the frequency of expression of the BCSC markers CD44, CD49F, P-cadherin, EPCAM, and ALDH1 the BR-BCSC signature in primary carcinomas vs. breast cancer brain metastases. Chi-square tests were performed to estimate the relationship between staining patterns of each BCSC marker in both primary and metastases series, Supplementary Table S6: Survival for the individual BCSC markers (CD44, CD49f, P-cadherin, EPCAM, and ALDH1), BR-BCSC signature, lymph node invasion, and combined lymph node status with BR-BCSC profile, Supplementary Table S7: BR-BCSC signature in primary breast cancer associates with clinical poor prognostic factors, Supplementary Table S8: BR-BCSC signature in the primary carcinomas associates with poor prognostic, basal-like, and glycolytic markers, Supplementary Table S9: Univariate and multivariate Cox proportional hazard analysis for the BR-BCSC signature in the primary carcinomas.

## Abbreviations

231.Br	MDA-MB-231.BR
231.Br.HER2	MB-231.BR.HER2 overexpressing cells
ALDH	Aldehyde dehydrogenase
BCSC	Breast cancer stem cells
BMFS	Brain metastases-free survival
BR-BCSC	Brain breast cancer stem cell signature
CAM	Chicken embryo chorioallantoic membrane model
CNS	Central nervous system
CSC	Cancer stem cells
DFS	Disease free survival
HER 2	Human epidermal growth factor receptor 2
MFE	Mammosphere forming efficiency
OS	Overall patient survival
REMARK	REporting recommendations for tumor MARKer prognostic studies
TNBC	Triple-negative breast cancer
