# Supplementary material for: BR-BCSC Signature: The Cancer Stem Cell Profile Enriched in Brain Metastases that Predicts a Worse Prognosis in Lymph Node-Positive Breast Cancer
Source: Cells. 2020 Nov 9;9(11):2442. doi: 10.3390/cells9112442 (PMC7695320; doi:10.3390/cells9112442)
Supplement: Supplementary file 1 [file cells-09-02442-s001.pdf]

cells

MDPI

BR-BCSC Signature: The Cancer Stem Cell Profile Enriched in Brain Metastases that Predicts a Worse Prognosis in Lymph Node-Positive Breast Cancer

Supplementary materials

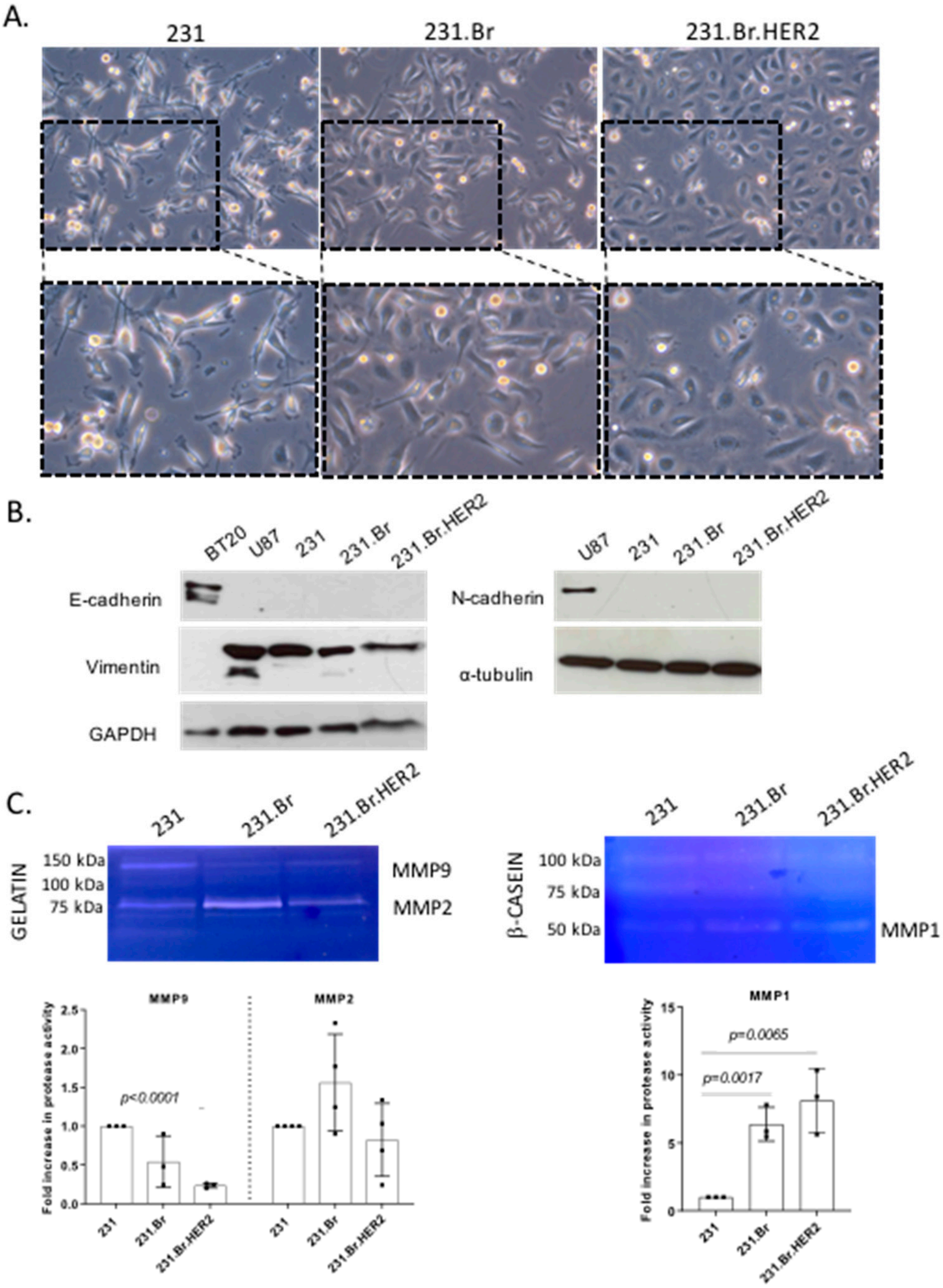

Supplementary Figure S1. Epithelial to mesenchymal transition phenotype. (**A**) Representative pictures of monolayer. (**B**) Western blot for EMT markers E-cadherin, N-cadherin and vimentin and the respective loading controls. (**C**) Gelatin and casein zymography for MMP9, MMP2 and MMP1 activity.

A.

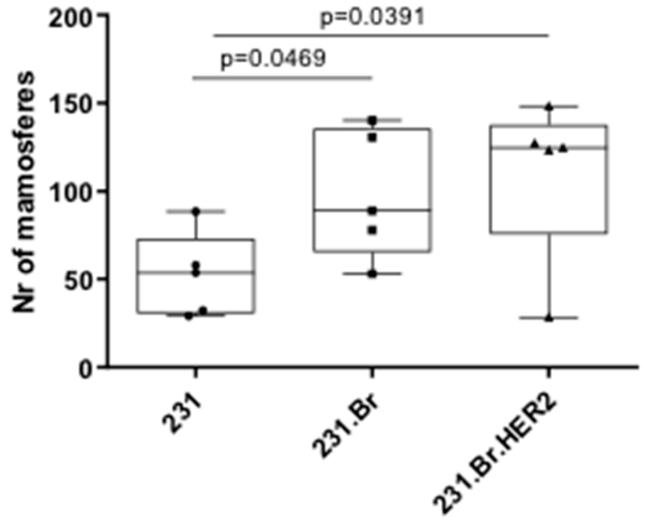

B.

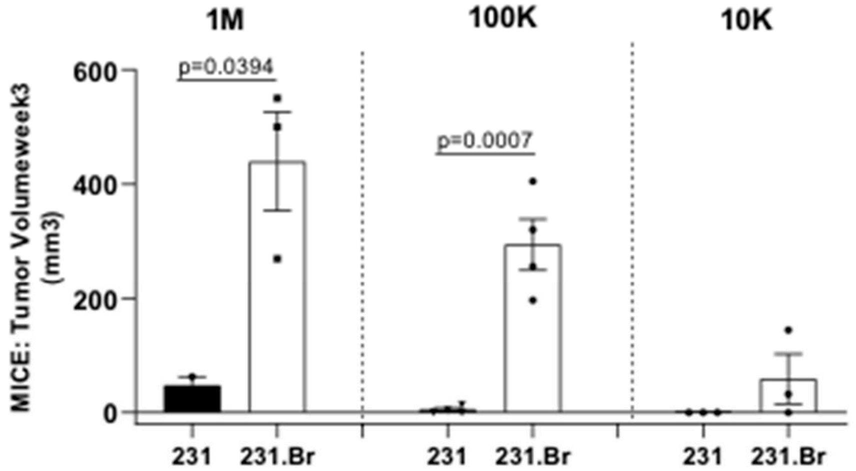

C.

Cell model	Cell number			Stem Cell frequency (ELDA)	p-value
	1M	100K	10K		
231	2/2 (100%)	2/4 (50%)	0/3 (0%)	1/157144	0.0041
231.Br	3/3 (100%)	4/4 (100%)	2/3 (66%)	1/9099	

Supplementary Figure S2. Stem cell activity. (**A**) Number of Mammospheres formation efficiency (MFE) in brain-tropic cells (231.Br and 231.Br.HER2) compared with parental cells (231). (**B**) Tumor size evaluation in a limiting dilution assay performed in the immunodeficient mice, by subcutaneous inoculation of 1M, 100K and 10K cell number per mice. (**C**) Number of tumors formed in in vivo nude mice, from 231 and 231.Br cell lines using serial dilutions of inoculated cells (limiting dilution assays).

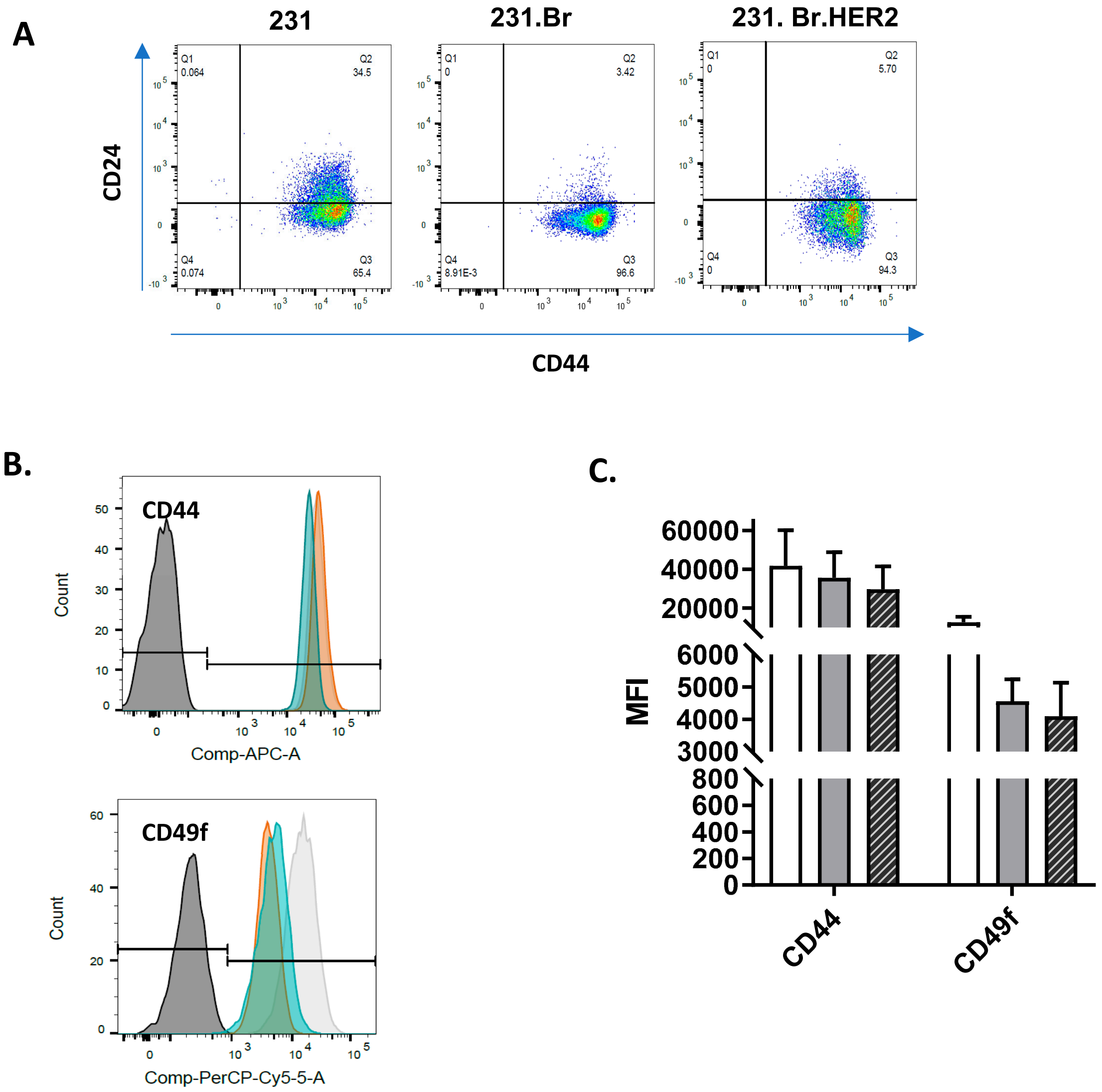

Supplementary Figure S3. Representative FACS analysis data. (**A**) Histograms for the differential flow cytometry profile of the stem cell marker combinations CD44/CD24. (**B**) histograms for CD44 and CD49f in the parental (soft grey) and the brain-tropic cells (231.Br in orange and 231.Br.HER2 in blue). (**C**) Mean Intensity fluorescence (MFI), measured by FACS, for CD44 and CD49f, in the different metastatic breast cancer cells.

Supplementary Table S1. Characterization and Clinico-pathological features of the primary breast cancer series.

**Database**	**Primary Tumor**	
Country	**Spain**	
Hospital	**Vigo**	
NR	**467**	
Type	retrospective	
Date	1978-1992	
Age at diagnosis	59,2 ± 13,29 (28-92)	
Follow-up duration	120 months	
DFS (months)	85,53 ± 43,92 (1-120)	
OS (months)	90,17 ± 40,744 (1-120)	
	**Frequency**	**Percentage (%)**

**Histological Grade**		
Grade 1	101	24,8
Grade 2	244	59,8
Grade 3	63	15,4
missing (*n* = 59)		
**Lymph Node Invasion**		
Negative	159	43,6
Positive	206	56,4
missing (*n* = 102)		
**Molecular subtype**		
Luminal	308	69,4
HER2+	68	15,3
TPN	68	15,3
missing (*n* = 23)		

Supplementary Table S2. Characterization and Clinico-pathological features of the two brain metastasis series (from Portugal and Brasil).

**Database**	**PT Series**		**BR Series**	
**Country**	Portugal		Brasil	
**Hospital**	CHLN		Barretos Cancer Hospital	
**Ethical approval**	01/2017/CEFCM		2.777.372	
**Nr of cases**	29		27	
**Type of database**	retrospective		retrospective	
**Years of diagnosis**	2009-2013		2012-2018	
**Age at diagnosis**	57,4 ± 11,6 (28-86)		48,9 ± 11,4 (26,5-70,8)	
**Follow-up duration**	120,0		120,0	
**BMFS (months)**	46,7 ± 42,0 (0-120)		28,2 ± 28,23 (0-110)	
**OS (months)**	61,6 ± 37,5 (1-120)		50,3 ± 32,3 (6-128)	
	**Portugal**		**Brasil**	
	**Frequency**	**Percentage (%)**	**Frequency**	**Percentage (%)**
**QT_Neo**				
No	0	0	16	59,3
Yes	29	100	11	40,7
**Stage**				
T1	3	10,3	5	18,5
T2	9	31,0	6	22,2
T3	6	20,7	5	18,5
T4	5	17,2	10	37,0
Und	6	20,7	1	3,7
**Lymph Node Invasion**				
Negative	12	41,4	8	29,6
Positive	11	37,9	18	66,7
Und	6	20,7	1	3,7
**Metastasis at diagnosis**				
Negative	19	65,5	21	77,8
Positive		0,0	6	22,2
Und	10	34,5	0	0,0
**Primary subtype**				
Luminal	14	48,3	11	40,7

HER2+	12	41,4	8	29,6
TPN	2	6,9	9	33,3
Und	5	17,2	1	3,7

Supplementary Table S3. Frequency of expression of the BCSC markers CD44, CD49F, P- cadherin, EPCAM and ALDH1 and the BR-BCSC signature in independent breast cancer brain metastases.

	**PT Series (*n* = *29*)**		**BR Series (*n* = *27*)**	
	**Frequency**	**Percentage (%)**	**Frequency**	**Percentage (%)**
**CD44**				
Negative	4	13,8	3	11,1
Positive	25	86,2	24	88,9
Missing (*n* = 0)				
**CD49F**				
Negative	13	44,8	7	25,9
Positive	16	55,2	20	74,1
Missing (*n* = 0)				
**P-cadherin**				
Negative	16	57,1	11	40,7
Positive	12	42,9	16	59,3
Missing (*n* = 1)				
**EPCAM**				
Negative	21	72,4	6	22,2
Positive	8	27,6	21	77,8
Missing (*n* = 0)	0	0,0	0	0,0
**ALDH1a1**				
Negative	24	92,3	20	74,1
Positive	2	7,7	7	25,9
Missing (*n* = 3)				
**BR-BCSC**				
0-2 BCSC	16	61,5	8	29,6
3-5 BCSC	10	38,5	19	70,4

Supplementary Table S4. Frequency of expression of the BCSC markers CD44, CD49F, P- cadherin, EPCAM and ALDH1 the BR-BCSC signature in primary carcinomas.

**Variable (*n* = *467*)**	**Frequency**	**Percentage (%)**
**CD44** [40]		
Negative	226	48,8
Positive	237	51,2
Missing (*n*= *4*)		
**CD49F** [23]		
Negative	378	88,5
Positive	49	11,5
Missing (*n* = *40*)		
**P-cadherin** [43]		
Negative	352	75,5
Positive	114	24,5
Missing (*n* = *1*)		
**EPCAM**		

Negative	383	91,0
Positive	38	9,0
Missing (*n*= *46*)		
**ALDH1a1** [40]		
Negative	451	96,8
Positive	15	3,2
Missing (*n*= *1*)		
**BR-BCSC**		
0-2 BCSC	409	90,7
3-5 BCSC	42	9,3
Missing (*n*= *16*)		

Supplementary Table S5. Contingency table with the frequency of expression of the BCSC markers CD44, CD49F, P- cadherin, EPCAM and ALDH1 the BR-BCSC signature in primary carcinomas vs Breast cancer brain metastasis. Chi-square tests was performed to estimate the relationship between staining patterns of each BCSC marker in both primary and metastasis series.

	**Primary Tumor (*n* = *467*)**		**Breast Cancer Brain Metastasis (*n* = *56*)**		
**Variable (*n* = *467*)**	**Frequency**	**Percentage (%)**	**Frequency**	**Percentage (%)**	***p*-Value**
**CD44**					
Negative	226	48,8	7	12.3	**<0.0001**
Positive	237	51,2	**50**	**87.7**	
**CD49f**					
Negative	378	88,5	19	33.3	**<0.0001**
Positive	49	11,5	**38**	**66.7**	
**P-cadherin**					
Negative	352	75,5	19	33.3	**<0.0001**
Positive	114	24,5	**38**	**66.7**	
**EPCAM**					
Negative	383	91	27	47.4	**<0.0001**
Positive	38	9	**30**	**52.6**	
**ALDH1a1**					
Negative	451	96,8	24	92.3	ns
Positive	15	3,2	2	7.7	
**BR-BCSC**					
0-2 BCSC	409	90,7	24	44.4	**<0.0001**
3-5 BCSC	42	9,3	**30**	**55.6**	

Supplementary Table S6. Survival for the individual BCSC markers (CD44, CD49f, P-cadherin, EPCAM and ALDH1), BR-BCSC signature, Lymph node invasion and combined lymph node status with BR-BCSC profile. Survival means were estimated using the Kaplan–Meier method and compared using the log-rank (Mantel-Cox) test. A significant level of 5% was considered. Missing cases were not considered for statistical analysis.

	**5-Year DFS**					**10-Year DFS**					**5-Year OS**					**10-Year OS**				
	**Mean**	**Std Dev**	**95% CI**		***p*-Value**	**Mean**	**Std Dev**	**95% CI**		***p*-Value**	**Mean**	**Std Dev**	**95% CI**		***p*-Value**	**Mean**	**Std Dev**	**95% CI**		***p*-Value**
			**Inf**	**Sup**				**Inf**	**Sup**				**Inf**	**Sup**				**Inf**	**Sup**	
**CD44**																				
Negative (*n* = 209)	**48,8**	1,3	46,3	51,4	0,557	**84,2**	3,1	78,2	90,2	0,174	**52,3**	1,0	50,3	54,3	0,849	**90,0**	2,8	84,6	95,4	0,272
Positive (*n* = 224)	**49,5**	1,2	47,1	51,9		**88,1**	2,9	82,4	93,8		**51,3**	1,1	49,1	53,5		**92,0**	2,8	86,6	97,4	
**CD49F**																				
Negative (*n* = 354)	**49,8**	1,0	47,9	51,7	0,479	**87,2**	2,3	82,7	91,7	0,535	**52,3**	0,8	50,8	53,9	0,28	**92,1**	2,1	88,0	96,3	0,447
Positive (*n* = 46)	**46,1**	3,2	39,8	52,4		**83,2**	7,0	69,4	96,9		**47,7**	2,9	42,0	53,5		**86,5**	6,8	73,1	99,9	
**P-cadherin**																				
Negative (*n* = 329)	**50,7**	1,0	48,8	52,6	**0,007**	**88,9**	2,3	84,3	93,4	**0,048**	**53,4**	0,8	51,9	54,9	**0,002**	**94,3**	2,1	90,1	98,4	**0,023**
Positive (*n* = 107)	**44,9**	2,0	41,0	48,9		**78,4**	4,6	69,4	87,5		**46,9**	1,9	43,3	50,5		**81,4**	4,5	72,7	90,2	
**EPCAM**																				
Negative (*n* = 357)	**49,3**	1,0	47,3	51,2	0,828	**86,4**	2,3	81,8	90,9	0,925	**51,6**	0,9	50,0	53,3	0,944	**91,2**	2,2	87,0	95,4	0,839
Positive (*n* = 35)	**48,9**	3,3	42,4	55,4		**87,3**	7,5	72,6	102,1		**51,8**	2,7	46,5	57,1		**91,8**	6,8	78,4	105,1	
**ALDH1**																				
Negative (*n* = 421)	**49,3**	0,9	47,5	51,1	0,441	**86,5**	2,1	82,4	90,7	0,792	**51,9**	0,8	50,4	53,5	0,083	**91,5**	2,0	87,6	95,4	0,507
Positive (*n* = 15)	**47,9**	4,1	39,8	56,0		**79,7**	11,4	57,4	101,9		**48,3**	4,1	40,3	56,2		**80,3**	11,2	58,2	102,3	
**BR-BCSC**																				

0-2 BSCS markers (*n* = 41)	**50,2**	0,9	48,4	52,0	**0,018**	**88,2**	2,2	83,9	92,5	0,108	**52,8**	0,8	51,3	54,3	**0,004**	**93,2**	2,0	89,3	97,1	0,077
3-5 BSCS markers (*n* = 15)	**43,6**	3,3	37,1	50,1		**75,4**	7,6	60,6	90,2		**45,1**	3,2	38,8	51,3		**78,7**	7,5	63,9	93,5	
**Lymph node invasion**																				
Negative (*n* = 157)	**55,0**	1,0	52,9	57,0	**<0,001**	**101,0**	2,8	95,6	106,5	**<0,001**	**56,4**	0,9	54,8	58,1	**<0,001**	**104,9**	2,5	100,0	109,7	**<0,001**
Positive (*n* = 198)	**44,8**	1,5	41,9	47,8		**75,9**	3,3	69,4	82,5		**48,7**	1,3	46,3	51,2		**82,5**	3,1	76,5	88,6	
**LN/BR-BCSC**																				
Negative/Negative (*n* = 136)	**55,4**	1,1	53,2	57,5		**102,3**	2,9	96,6	107,9		**56,9**	0,9	55,2	58,6		**106,3**	2,5	101,3	111,3	
Negative/Positive (*n* = 17)	**53,9**	3,3	47,6	60,3	**<0,001**	**97,2**	8,9	79,8	114,7	**<0,001**	**54,7**	2,9	49,0	60,4	**<0,001**	**100,1**	8,6	83,2	116,9	**<0,001**
Positive/Negative (*n* = 174)	**46,3**	1,5	43,3	49,3		**79,0**	3,5	72,1	85,9		**50,4**	1,2	48,0	52,8		**86,2**	3,2	80,0	92,4	
Positive/Positive (*n* = 17)	**36,9**	5,5	26,2	47,6		**59,8**	11,8	36,7	82,9		**37,2**	5,4	26,6	47,7		**60,4**	11,8	37,3	83,5	

Supplementary Table S7. BR-BCSC signature in primary breast cancer associates with clinical poor prognostic factors. Contingency tables and chi-square test were used to estimate the relationship between BR-BCSC signature with the classical clinic-pathological parameters such as tumor size, Grade, Lymph nodes invasion. *P* values <0.05 were considered statistically significant. The bolded values refer to significant results.

	**BR-BCSC Signature**				
	**0-2 SC Markers**		**3-5 SC Markerrs**		***p*-Value**
	**Frequency**	**Percentage (%)**	**Frequency**	**Percentage (%)**	
Tumor size					
T1: <2cm	99	27.6	1	2.6	
T2: 2-5 cm	211	58.8	26	68.4	*0.0001*
T3: >5cm	49	13.6	11	28.9	
Missing (*n*= *69*)					
Grade					
1	77	19.8	4	9.5	
2	124	32.0	6	14.3	*0.0003*
3	187	48.2	32	76.2	
Missing (*n*= *37*)					
Lymph node invasion					
Negative	138	43.4	17	48.6	
Positive	180	27.0	18	11.4	0.593
Missing (*n*= *114*)					

Supplementary Table S8. BR-BCSC signature in the primary carcinomas associates with poor prognostic, basal-like and glycolytic markers. Contingency tables and chi-square test were used to estimate the relationship between BR-BCSC signature with the molecular markers ER, PR, HER2, ki-67, EGFR, vimentin, CK5, CK14, p63, GLUT1 and CAIX. *P* values <0.05 were considered statistically significant. The bolded values refer to significant results.

	**BR-BCSC Signature**				
	**0-2 SC Markers**		**3-5 SC Markers**		***p*-Value**
	**Frequency**	**Percentage (%)**	**Frequency**	**Percentage (%)**	
**ER**					
negative	113	27.8	**34**	**81.0**	**<0.0001**
positive	294	72.2	8	19	
Missing (*n* = *18*)					
**PR**					
negative	199	48.8	**31**	**73.8**	**0.0002**
positive	209	51.2	11	26.2	
Missing (*n* = *17*)					
**HER2**					
negative	349	86.2	34	81.0	ns
positive	56	13.8	8	19.0	
Missing (*n* = *20*)					
**kI67**					
0	20	4.9	1	2.4	**<0.0001**
<6.4%	275	67.2	13	31.0	
>6.4%	114	27.9	**28**	**66.7**	
Missing (*n* = *16*)					
**EGFR**					

negative	397	97.1	34	81.0	**<0.0001**
positive	12	2.9	**8**	**19.0**	
Missing (*n* = *16*)					
**Vimentin**					
negative	348	86.8	22	52.4	**<0.0001**
positive	53	13.2	**20**	**47.6**	
Missing (*n* = *24*)					
**CK5**					
negative	367	90.0	22	52.4	**<0.0001**
positive	41	10.0	**20**	**47.6**	
Missing (*n* = *17*)					
**CK14**					
negative	392	96.1	36	85.7	**0.011**
positive	16	3.9	**6**	**14.3**	
Missing (*n* = *17*)					
**P63**					
negative	371	94.9	35	85.4	**0.027**
positive	20	5.1	**6**	**14.6**	
Missing (*n* = *35*)					
**GLUT1**					
negative	322	85.4	23	54.8	**<0.0001**
positive	55	14.6	**19**	**45.2**	
Missing (*n* = *48*)					
**CAIX**					
negative	162	42.9	9	22.5	**0.017**
positive	216	57.1	**31**	**77.5**	
Missing (*n* = *49*)					

Supplementary Table S9. Univariate and Multivariate Cox proportional hazard analysis for the BR-BCSC signature in the primary carcinomas. This analysis allows risk prediction (hazard ratios and the corresponding 95% confidence interval) for DFS and OS of breast cancer patients. A significant level of 5% was considered. Missing cases were not considered for statistical analysis. The multivariate COX regression analysis included the effects lymph node invasion, histological grade and tumor size.

PRIMARY TUMOR	Disease-Free Survival								Overall survival							
	5-Year Survival				*10-Year Survival*				*5-Year Survival*				*10-Year Survival*			
	HR	*95% CI*		*p-Value*	*HR*	*95% CI*		*p-Value*	*HR*	*95% CI*		*p-Value*	*HR*	*95% CI*		*p-Value*
		**Inferior**	**Superior**			**Inferior**	**Superior**			**Inferior**	**Superior**			**Inferior**	**Superior**	
**LN/BR-BCSC**																
Negative/Negative (*n* = 136, ref.)	1,00			—	1,00			—	1,00			—	1,00			—
Negative/Positive (*n* = 17)	1,62	0,55	4,75	0,38	1,57	0,70	3,52	0,27	2,08	0,69	6,21	0,19	1,68	0,70	4,04	0,25
Positive/Negative (*n* = 174)	2,94	1,78	4,85	0,00	2,36	1,61	3,46	0,00	3,13	1,79	5,45	0,00	2,60	1,71	3,95	0,00
Positive/Positive (*n* = 17)	5,71	2,67	12,21	0,00	3,80	1,94	7,46	0,00	7,89	3,58	17,42	0,00	5,10	2,55	10,19	0,00
**LN/BR-BCSC**																
Negative/Negative (*n* = 136, ref.)	1,00			—	1,00			—	1,00			—	1,00			—
Negative/Positive (*n* = 17)	1,16	0,39	3,49	0,79	1,20	0,52	2,74	0,67	1,35	0,44	4,12	0,60	1,25	0,51	3,07	0,62
Positive/Negative (*n* = 174)	2,33	1,35	4,02	0,00	1,89	1,25	2,85	0,00	2,15	1,19	3,88	0,01	1,98	1,27	3,10	0,00
Positive/Positive (*n* = 17)	3,49	1,56	7,83	0,00	2,34	1,15	4,73	0,02	4,13	1,80	9,51	0,00	3,04	1,47	6,29	0,00
**Histological grade**																
Grade I (*n* = 54, ref.)	1,00			—	1,00			—	1,00			—	1,00			—
Grade II (*n* = 94)	0,91	0,39	2,13	0,84	1,21	0,63	2,32	0,56	1,11	0,43	2,86	0,83	1,19	0,61	2,34	0,61
Grade III (*n* = 175)	1,85	0,87	3,92	0,11	1,85	1,02	3,37	0,04	2,00	0,85	4,71	0,11	1,62	0,87	3,02	0,13
**Tumor size**																
T1: < 2 cm (*n* = 82, ref.)	1,00			—	1,00			—	1,00			—	1,00			—
T2: 2–5 cm (*n* = 189)	1,86	0,93	3,73	0,08	1,65	0,98	2,77	0,06	2,70	1,13	6,43	0,03	1,88	1,06	3,35	0,03
T3: > 5 cm (*n* = 52)	3,21	1,49	6,91	0,00	2,99	1,66	5,39	0,00	5,11	2,02	12,92	0,00	3,45	1,81	6,59	0,00

Survival HR were evaluated according with the BR-BCSC signature in primary tumors and breast cancer brain metastases(univariate analysis). The multivariate COX regression analysis included the effects histological grade, tumor size, and lymph node invasion. Missing cases were not considered for statistical analysis.
